# A different vision of dyslexia: Local precedence on global perception

**DOI:** 10.1038/s41598-017-17626-1

**Published:** 2017-12-12

**Authors:** Sandro Franceschini, Sara Bertoni, Tiziana Gianesini, Simone Gori, Andrea Facoetti

**Affiliations:** 1 Developmental and Cognitive Neuroscience Lab, Department of General Psychology, University of Padua, Padova, 35131 Italy; 2 Child Psychopathology Unit, Scientific Institute “E. Medea”, Bosisio Parini, Lecco, 23842 Italy; 3 Neurorehabilitation Unit, Down syndrome association, Verona, 37142 Italy; 4 Department of Human and Social Sciences, University of Bergamo, Bergamo, 24129 Italy

## Abstract

Individuals perceive the wor(l)d hierarchically. Firsty, the global visual scene is processed by the right hemisphere, and later, the local features are perceived by the left hemisphere. Based on this hierarchical analysis, humans evolved unique communication ability: reading. However, for about 10% of people reading acquisition is extremely difficult, they are affected by a heritable neurodevelopmental disorder called dyslexia. Differences in perceiving the wor(l)d might be one of the causes of reading disabilities. Here we show multiple causal links between the global before local perception and learning to read. Five behavioral experiments in 353 children reveal that: (i) a local before global perception characterizes three independent groups of unselected children with dyslexia; (ii) two global before local perception trainings improve reading skills in children with dyslexia; and stringently (iii) pre-reading local before global perception longitudinally predicts future poor readers. Challenging the uni-causal and left-lateralized phonological explanation of dyslexia, our results demonstrate that learning to read depends also on an efficient right neural network for the global analysis of the visual scene. These results provide new insights in learning strategies and pave the way for early identification and possible prevention programs.

## Introduction

Dyslexia is defined by the failure in developing an accurate and fluent reading in presence of normal intelligence, teaching, environmental support, relative treatment resistance, and lack of sensorial deficits^1,2^. Learning to read involves multiple linguistic^3–5^, visual^6–8^ and attentional^9–13^ processes. However, the current view claims that dyslexia is an impairment in phonological awareness, which is the ability of the left temporo-parietal network to perceive and manipulate the sounds of spoken words^2–5,14^. While the relevance of phonological awareness deficits should not be minimized, there is substantial evidence that other factors are also involved in dyslexia^5–16^, the most frequent neurodevelopmental disorder. Clinical heterogeneity and high level of comorbidity with attentional deficit and hyperactivity, and autism spectrum disorders, characterize dyslexia^1,17–21^.

Neuropsychological, psychophysical, electrophysiological and functional neuroimaging studies have suggested that the right temporo-parietal junction plays a key role in low spatial frequency processing (global perception), while the homologous area in the left hemisphere specifically processes the high spatial frequencies (local details)^22–27^. Navon^22^ describes global precedence on local perception as an inherent property of the human visual system that usually could not be skipped^23^.

During the orthographic processing - before phonological mapping - perception of the global scene is a useful device for narrowing down the range of candidates in accounting for a certain local region and their location assignments^10,11,22,23,28^. Later, sequential scanning of individual letters inside fixation periods is also necessary for effective letters identification^11,12,28^.

Reversing the global to local world perception has been found to be associated with unusual and extraordinary performance in local features extraction in several neurodevelopmental disorders often in comorbidity with dyslexia^17–21^.

**Figure 1:**
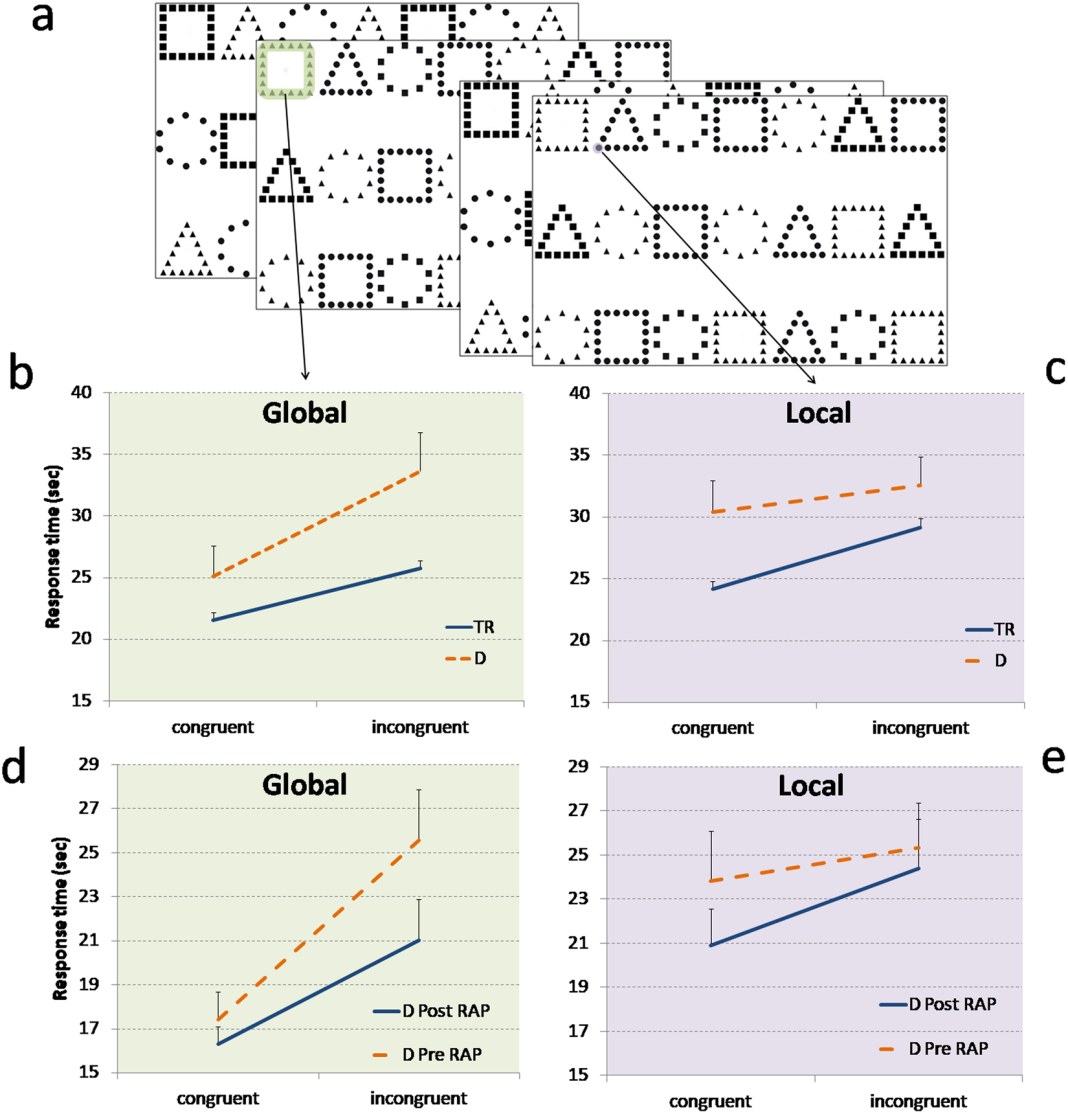
Paper and pencil Navon multiple stimuli naming task and results of Experiments 1 and 2. (**a**) In the global tasks, children were invited to name aloud the larger figures, independently from the local figures. In contrast, in the local tasks, children were invited to name aloud the smaller figures, independently from the global figures. Global and local figures could be congruent (for example a square composed by squares) or incongruent (a square composed by triangles). (**b**,**c**) Experiment 1. An unselected group of children with dyslexia (D) showed greater local interference than TR in global task (**b**), and lower global interference in local task **(c)**. (**d**,**e**) Experiment 2. An unselected group of children with dyslexia, after the reading acceleration program (RAP) training, showed a significant reduction of local interference effect in global task (**d**), and a significant condition (incongruent vs. congruent) effect in local task (**e**). Data are mean ± standard errors (SEM).

**Figure 2:**
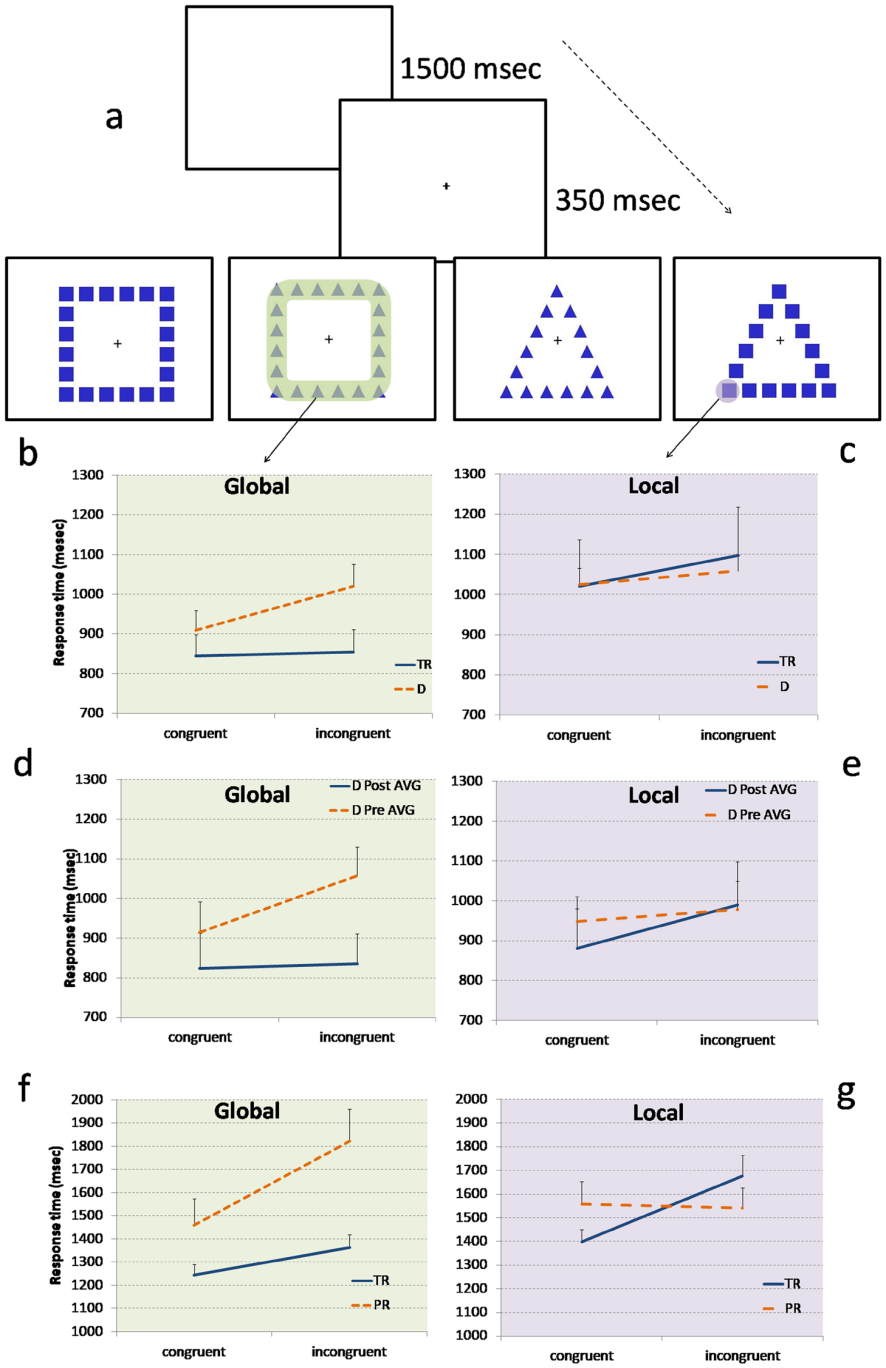
Computerized Navon task and results of Experiments 3, 4 and 5. (**a**) In the global task, children were invited to discriminate the larger figures (triangle or square) by pressing two different buttons of keyboard, independently from the local figures. In contrast, in the local task, children were invited to discriminate the smaller figures, independently from the global figures. Global and local figures could be congruent (for example a square of squares) or incongruent (a square of triangles). (**b**,**c**) Experiment 3. An unselected group of children with dyslexia (D) showed greater local interference than TR in global task (**b**) and lower global interference in the local task **(c)**. (**d**,**e**) A subgroup of children with dyslexia of Experiment 3 were treated with action video game (AVG) and non action video game training in Experiment 4. Only after the AVG training children with dyslexia showed a significant reduction of local interference effect in the global task (**d**) and a significant increase of global interference effect in the local task (**e**). (**f**,**g**) Experiment 5. In an unselected group of prereading children, global and local visual perception at kindergarten were measured and their reading abilities at the end of the first grade were longitudinally investigated. Future PR showed already at pre-reading stage a greater local interference than future TR in the global task (**f**) and lower global interference in the local task **(g)**. Data are mean ± standard errors (SEM).

Here, we employ a comprehensive approach incorporating all causal methods to test the relationship between dyslexia and local before global perception, assessed by performance on paper and pencil rapid automatized naming (RAN; Fig. 1a) or computerized Navon tasks (Fig. 2a). These methods are: (1) comparison with typically reading controls (see Experiments 1 and 3); (2) remediation studies, in which global before local perception (by reading program and non-reading action video game, see Experiments 2 and 4, respectively) is specifically trained and the subsequent effect on reading improvements is measured, and stringently; (3) a longitudinal approach where global before local perception is measured in pre-readers and its predictability with future reading development is prospectively investigated (see Experiment 5).

Dyslexia is characterized by a reduced reading experience that could impair the letter processing^5,14,28^.Consequently, we employed geometric figures composed by smaller figures instead of the typical letters composed by smaller letters^17,19,20,22^.

## Results

### Experiment 1: Global visual perception in children with dyslexia by using a paper and pencil Navon task

To investigate local and global perception in a paper and pencil Navon task, the corrected response times were analyzed with a mixed analysis of variance (ANOVA). The Navon task was administered to two unselected groups of children from primary school, with and without dyslexia. Whether a different visual perception characterize the neurodevelopmental disorder of reading acquisition, a reversed global precedence perception, i.e. local before global perception will be expected in our sample of children with dyslexia.

Response times (in sec) in the Navon stimuli task were analyzed by a mixed ANOVA with a 2 × 2 × 2 design. The two within-subject factors were condition (congruent and incongruent) and task (global and local), while the between-subject factor was group (children with dyslexia and typical readers, TR). Crucially for our hypothesis, condition × task × group interaction was significant (F_(1,177)_ = 10.472, p = 0.001, η^2^ = 0.056), indicating that the two groups showed a different condition effect in the two tasks (Fig. 1b,c).

Within-subject planned comparisons on the condition effect (incongruent vs. congruent) were significant in global (t_(161)_ = −10.67, p = 0.0001, Cohen’s d = 0.84, scaled JZS Bayes factor with r = 0.5 (B_01_) > 30) and local (t_(161)_ = −10.289, p = 0.0001, Cohen’s d = 0.81 B_01_ > 30) task in TR group, whereas this effect was significant in global (t_(16)_ = −3.522, p = 0.003, Cohen’s d = 0.85, B_01_ = 14.8), but not in local task (t_(16)_ = −1.045, p = 0.312, Cohen’s d = 0.25, B_01_ in favor of the null = 1.99) in the group with dyslexia.

Between-subject planned comparisons showed that in the global task, the condition effect was significantly increased in the group with dyslexia (mean = 8.55 sec, SD = 10.01) in comparison to TR group (mean = 4.21 sec, SD = 5.02; t_(177)_ = −3.014, p = 0.001, Cohen’s d = 0.58, B_01_ = 11.82). In contrast, in the local task, the condition effect was significantly decreased in the group with dyslexia (mean = 2.16 sec, SD = 8.55) in comparison to TR group (mean = 4.96 sec, SD = 6.14; t_(177)_ = 1.715, p = 0.044, Cohen’s d = 0.38, B_01_ = 1.04; see SI for the other results).

### Experiment 2: Global visual perception in children with dyslexia after a visual treatment

To demonstrate the possible causal role of global before local perception on reading skills, in an independent and unselected sample of children with dyslexia (n = 13), we used a visual attentional training, i.e. reading acceleration program, (RAP)^29^. This training forced the rapid global processing of written sentences, without any explicit intervention on phonological processing.

To evaluate the influence of training on reading abilities, we used a within-subjects experimental design. Global and local perception in the Navon task, auditory-phonological and reading abilities were tested three times at identical time interval (T1, T2 and T3). The RAP training was applied to the same children between T2 and T3 (training phase), whereas no-training was applied between T1 and T2 (no-training phase). Perceptual, phonological and reading skills were analyzed by a series of mixed ANOVA. After the RAP training, in addition to an improvement in the reading and auditory-phonological skills, a reduction of local processing in the global task (i.e., decreased local before global perception) was also expected.

The training effect on the response time in the Navon multiple stimuli naming task was evaluated by a mixed ANOVA with 3 within-subject factors: time (T1, T2 and T3) × condition (congruent and incongruent) × task (global and local). Crucially for our hypothesis, the time × condition × task interaction was significant (F_(2,24)_ = 3.723, p = 0.039 η^2^ = 0.237). To better understand this interaction, we analyzed the data obtained in T1 and T2 (no-training phase), and in T2 and T3 (training phase) in two separate ANOVAs. In the first ANOVA on no-training phase, only the condition × task interaction was significant (F_(1,12)_ = 14.869, p = 0.002 η^2^ = 0.553; not significant time main effect: F_(1,12)_ = 4.4, p = 0.06 η^2^ = 0.268). In the second ANOVA on training phase, the time main effect was significant (F_(1,12)_ = 9.57, p = 0.009 η^2^ = 0.443). Crucially, the time × condition × task interaction was also significant (F_(1,12)_ = 7.061, p = 0.021, η^2^ = 0.37), indicating a different condition effect in the two tasks induced by RAP training. Before RAP training (T2) paired sample t-tests showed a significant condition effect (i.e., incongruent vs. congruent) only in global task (t_(12)_ = 3.268, p = 0.007, Cohen’s d = 1.27, B_01_ = 7.64; local task t_(12)_ = 0.82, p = 0.428, Cohen’s d = 0.19, B_01_ in favor of the null = 2.14), confirming the results found in Experiment 1 for the children with dyslexia. In contrast, after RAP training (T3), the condition effect was significant both in global (t_(12)_ = 3.352, p = 0.006, Cohen’s d = 0.98, B_01_ = 8.61) and local task (t_(12)_ = 2.623, p = 0.022, Cohen’s d = 0.49, B_01_ = 3.12; Fig. 1d,e), indicating a similar pattern of results shown in Experiment 1 for TR (Fig. 1b,c). In addition, paired sample t-tests showed that after the RAP training children with dyslexia significantly reduced the response time only in the incongruent global condition (t_(12)_ = 2.193, p = 0.049, Cohen’s d = 0.61, B_01_ = 1.78; congruent global condition t_(12)_ = 1.04, p = 0.319, Cohen’s d = 0.30,, B_01_ in favor of the null = 1.84 congruent local condition t_(12)_ = 1.771, p = 0.102, Cohen’s d = 0.42, B_01_ = 1.08 incongruent local condition t_(12)_ = 0.766, p = 0.458, Cohen’s d = 0.12, B_01_ in favor of the null = 2.21), suggesting that RAP training reduced the local before global perception in children with dyslexia.

Children with dyslexia improved significantly their text reading speed (T2 mean = 301 sec, SD = 213 vs. T3 mean = 228 sec, SD = 105; t_(12)_ = 2.257 p = 0.043, Cohen’s d = 0.46 B_01_ = 1.93) and phonological decoding speed (T2 mean = 223 sec, SD = 84 vs. T3 mean = 188 sec, SD = 47; t_(12)_ = 2.349 p = 0.037, Cohen’s d = 0.53 B_01_ = 2.17) only after RAP training (see SI for further details).

The accuracy in pseudowords repetition (see SI for details) was analyzed by a within subjects ANOVA (time: T1, T2 and T3). Results revealed a significant effect of time (F_(1,12)_ = 7.779, p = 0.002, η^2^ = 0.393). Paired sample t-tests showed that the difference in accuracy between T1 (mean accuracy = 29.69, SD = 5.57) and T2 (mean accuracy = 31.08, SD = 5.44) was not significant (t_(12)_ = 1.322, p = 0.211, Cohen’s d = 0.25, B_01_ = in favor of the null = 1.46), whereas the accuracy improvements between T2 and T3 (mean = 33.69, SD = 3.99) and T1 and T3 were both significant (t_(12)_ = 2.601, p = 0.023, Cohen’s d = 0.55, B_01_ = 3.02 and t_(12)_ = 4.129, p = 0.001, Cohen’s d = 0.84, B_01_ = 26.29, respectively), showing that RAP training significantly increased also the auditory-phonological skills involved in pseudowords repetition task.

### Experiment 3: Global visual perception in children with dyslexia by using a computerized Navon task

Similarly to Experiment 1, a Navon task was administered to two unselected groups of children from primary school, with and without dyslexia. In this experiment, a computerized Navon task was used to exclude the effects driven by the multiple stimuli presentation and verbal response (i.e., perceptual load, serial attentional processing and phonological mapping) involved in our previous paper and pencil Navon task (Experiments 1 and 2). Response times in global and local tasks were analyzed with a mixed ANOVA. As found in Experiment 1, an atypical local before global perception was expected also in this new and unselected sample of children with dyslexia.

In Experiment 3, a new group of children with dyslexia (n = 32) and a new group of TR (n = 15) were tested. Response times (in msec) in the computerized Navon task (Fig. 2a) were analyzed by means of a mixed ANOVA with a 2 × 2 × 2 design. The two within-subject factors were condition (congruent and incongruent) and task (global and local), while the between-subject factor was group (children with dyslexia and TR).

Crucially for our hypothesis, condition × task × group interaction was significant (F_(1,45)_ = 5.697, p = 0.021, η^2^ = 0.112), indicating that the two groups showed a different condition effect in the two tasks (Fig. 2b,c).

Since the main results were found in global perception, we collected further 17 TR children only in global task (n = 32) to balance the different sample size of two groups originally studied (see SI for details).

Within-subject planned comparisons on the condition effect (incongruent vs. congruent) were not significant in global (t_(31)_ = 0.667, p = 0.51, Cohen’s d = 0.03, B_01_in favor of the null = 3.24), but were significant in local (t_(14)_ = 3.873, p = 0.002, Cohen’s d = 0.17, B_01_ = 22.55) task in TR group, whereas this effect was significant in global (t_(31)_ = 6.599, p = 0.0001, Cohen’s d = 0.37, B_01_ > 30), but not in local task (t_(31)_ = 1.719, p = 0.096, Cohen’s d = 0.15, B_01_ in favor of the null = 1.15) in children with dyslexia. Moreover, in global task, the condition effect was significantly increased in children with dyslexia (mean = 111 msec, SD = 95) in comparison to TR group (mean = 10 msec, SD = 88; t_(62)_ = 4.383, p = 0.0001, Cohen’s d = 1.10, B_01_ > 30). In contrast, in local task, the condition effect was decreased in children with dyslexia (mean = 33 msec, SD = 109) in comparison to TR group (mean = 77 msec, SD = 77; but t_(45)_ = −1.402, p = 0.168, Cohen’s d = 0.47, B_01_in favor of the null = 1.28) (see SI for the other results).

### Experiment 4: Global visual perception in children with dyslexia after an action video game training

In this experiment we investigated the effect of an action video game (AVG) training on global and local perception, and reading skills. A sub-group of children with dyslexia of Experiment 3 (n = 14) was randomly assigned to an AVG (n = 7) or a non action video game (NAVG, n = 7) training^8,13,30–33^. Response times in computerized Navon task and reading skills (time and errors) were analyzed with mixed ANOVA design. In addition to an improvement in the reading speed, a specific reduction of local before global perception was also expected.

Response times (in msec) in the global and local computerized Navon task (Fig. 2a) were analyzed by means of two mixed ANOVAs with a 2 times (T1 = before and T2 = after) × 2 conditions (congruent and incongruent) design for each treated group.

In the AVG group, in global task, main effect of condition was significant (F_(1,6)_ = 11.28, p = 0.015, η^2^ = 0.653). Crucially for our hypothesis, time × condition interaction was also significant (F_(1,6)_ = 9.379, p = 0.022, η^2^ = 0.61). Planned comparison showed that AVG group presented a significant response times reduction in incongruent condition (t_(6)_ = 2.521, p = 0.045, Cohen’s d = 1.12, B_01_ = 2.12; Fig. 2d). The same ANOVA on NAVG did not show any significant effect (time × condition effect F_(1,6)_ = 0.180, p = 0.686, η^2^ = 0.029; see Fig. 1SI).

In local task, both groups showed only a main effect of condition (AVG: F_(1,6)_ = 6.921, p = 0.039, η^2^ = 0.536; NAVG: F_(1,6)_ = 9.959, p = 0.02, η^2^ = 0.624). Planned comparison driven by our previous results of Experiment 2, showed that in children with dyslexia, the condition effect (incongruent vs. congruent) became significant only after AVG treatment (t_(6)_ = 3.264 p = 0.017, Cohen’s d = 0.40, B_01_ = 4.06 Fig. 2e, whereas after NAVG t_(6)_ = 1.472 p = 0.19, Cohen’s d = 0.27, B_01_in favor of the null = 1.15; see Fig. 1SI).

Reading speed (syllables per second) improvement was evaluated in AVG and NAVG groups by two separate ANOVAs 2 times (T1 = before and T2 = after) × 3 tasks (words text, pseudowords lists and pseudowords texts). Results showed a significant main effect of time (F_(1,6)_ = 7.78, p = 0.032 η^2^ = 0.565; T1 mean = 1.59 SD = 0.41, T2 mean = 1.86, SD = 0.49) only in the AVG training group (NAVG time effect F_(1,6)_ = 1.097, p = 0.335 η^2^ = 0.155 T1 mean = 1.29 SD = 0.73, T2 mean = 1.37, SD = 0.65). The same ANOVAs considering as dependent variable the number of errors, did not show any significant effect (AVG time effect F_(1,6)_ = 1.931, p = 0.214 η^2^ = 0.243; T1 mean = 4.48 SD = 2.99, T2 mean = 4.21, SD = 3.09; NAVG time effect F_(1,6)_ = 0.692, p = 0.437 η^2^ = 0.103; T1 mean = 7.02 SD = 4.68, T2 mean = 6.99, SD = 3.42). The reading improvements after the AVG training were characterized by the increased reading speed without any cost in accuracy^8,13,33^ and this result is in agreement with the improved speed of processing already found associated with AVG^32^.

### Experiment 5: Pre-reading global visual perception in future children with reading disorders

In Experiment 5, we longitudinally investigated the causal link between global and local perception and learning to read, testing 96 pre-literate children. We divided our pre-reading sample in future poor readers (PR, n = 14) and TR (n = 68) on the basis of their future standardized reading performance at the end of grade 1^12^. A child was assigned to the PR group if her/his z score for average fluency and accuracy text reading was below −1.5 SDs. All children who did not meet the criterion for inclusion in the PR group were assigned to the TR group (see SI for further details). Accuracy and reaction times in the computerized Navon task were analyzed with two mixed ANOVAs. We predicted a selective local before global perception at pre-reading stage only in future PR children.

Accuracy and response times (in msec) in the computerized Navon task were analyzed by two ANOVAs with a 2 × 2 × 2 design. The two within-subject factors were condition (congruent and incongruent) and task (global and local) and the between-subject factor was group (PR and TR). Crucially for our causal hypothesis, the condition × task × group interaction was significant only on response times ANOVA: F_(1,80)_ = 11.55, p = 0.001 η^2^ = 0.126 (Fig. 2f,g) (see also SI).

Within-subject planned comparisons on the condition effect (incongruent vs. congruent) showed a significant effect both in global (t_(67)_ = 3.276, p = 0.002, Cohen’s d = 0.28, B_01_ = 18.78) and in local (t_(67)_ = 5.203, p = 0.0001, Cohen’s d = 0.48, B_01_ > 30) tasks in future TR group, whereas in future PR group this effect was significant in global (t_(13)_ = 2.964, p = 0.011, Cohen’s d = 0.76, B_01_ = 5.22), but not in local task (t_(13)_ = 0.271, p = 0.791, Cohen’s d = 0.05, B_01_ in favor of the null = 2.75). The TR group showed a significantly greater condition effect in local than in global task (t_(67)_ = 2.428, p = 0.018, Cohen’s d = 0.43, B_01_ = 2.51), whereas the PR group showed a significantly greater condition effect in global than in local task (t_(13)_ = 2.623, p = 0.021, Cohen’s d = 1.08, B_01_ = 3.21).

Between-subject planned comparisons showed that in the global task, the condition effect was significantly stronger in PR (mean = 362 msec, SD = 458) in comparison to TR group (mean = 118 msec, SD = 298; t_(80)_ = 2.525, p = 0.014, Cohen’s d = 0.65, B_01_ = 3.82). In contrast, in the local task the condition effect was present in the TR group (mean = 277 msec, SD = 440) that showed a greater condition effect in comparison to the PR group (mean = −17 msec, SD = 245; t_(80)_ = 2.427, p = 0.017, Cohen’s d = 0.86, B_01_ = 3.2).

In addition, paired-sample t-tests revealed that the two groups significantly differed only in the global incongruent condition (t_(80)_ = 3.304, p = 0.001, Cohen’s d = 0.93, B_01_ = 20.57).

After we established that future PR, at the pre-reading stage, already showed a local before global visual perception, we further investigated the causal link between individual measures of neurocognitive functioning at T1 (kindergarten) and reading emergence (T2 = grade 1), across our entire sample of children (n = 82), independently of our a priori group classification of reading disorder. Using two five blocks fixed entry linear regression analysis, we showed that after controlling for chronological age, performance IQ (i.e., Block Design standard score)^34^, auditory-phonological (errors in the syllabic blending)^35^ and cross-modal mapping (i.e., speed in sec in the RAN of colours)^12^ skills, only the global task condition effect (incongruent vs. congruent) measured at pre-reading stage predicted a significant unique variance (R^2^ = 0.07, p = 0.017) of future text reading skills (mean between speed and accuracy z scores) in T2 (see SI for further results).

## Discussion

In Experiment 1, we studied the typical global before local perception in an unselected group of children with dyslexia and in TR of primary school. In comparison with TR, children with dyslexia showed no interference from the global information during the rapid naming in the local task (Fig. 1c). In contrast to TR performance, children with dyslexia presented a larger interference of the local incongruent feature during the rapid naming in the global perception task (Fig. 1b). These results demonstrate that children with dyslexia present a local before global perception.

However, the cross-sectional design used in this experiment was not useful to disentangle the causal relationship between the observed local before global perception and reading difficulties^5^. Poor global visual perception development could be a simple consequence of their reduced reading experience^14^.

In Experiment 2, a training design was employed in an independent and unselected sample of children with dyslexia to demonstrate the possible causal role of the typical global before local perception on efficient reading skills^5^. We used a visual attentional training (i.e., reading acceleration program, RAP) that involve a silent sentence reading in a multi-session procedure, with individually set time constraints (letter-by-letter masking)^29^. After this training, children with dyslexia showed a reduction of local interference in the global task (Fig. 1d) and better words and pseudowords reading performance. The training also produced an improvement in pseudowords repetition. In sum, children with dyslexia improved their reading and reading-related skills together with global before local perception.

The results obtained in Experiments 1 and 2 might still have been influenced by several aspects involved in our rapid multiple stimuli naming task. In particular, both attentional^10–13^ and phonological aspects^3,5^ are involved during this Navon task. Moreover, although the training improves the global before local perception without any direct auditory-phonological stimulation, the consequent reading improvement could also be due to a different cause. The RAP training^29^ might improve the letter-to-speech sound integration, which should result in better reading performance^14^. This could be also an alternative explanation of the auditory-phonological improvement observed in pseudowords repetition.

In Experiments 3, 4 and 5, other two independent and unselected samples of children were tested by a computerized version of Navon task (Fig. 2a). This Navon task did not involve the presence of more than one target at a time, reducing the possible role of perceptual load and serial attentional processing dysfunctions^7,12,13^. In addition, there was no involvement of speech sound processing in response measurement, excluding the influence of other neurocognitive functions known to be predictive of future reading abilities^5,12^.

In Experiment 3, a new group of children with dyslexia and a new group of TR were tested. Contrary to the typical global before local perception found in TR^22^, children with dyslexia showed greater local interference in the global task in comparison to the global interference during the local task. Moreover, children with dyslexia showed a greater local interference effect than the TR in the global incongruent condition, confirming that they present a local before global perception even when possible perceptual, attentional and linguistic effects were now excluded (Fig. 2b,c).

In Experiment 4, children with dyslexia were randomly assigned to an AVG or a NAVG training^8,13,30,33^. AVGs share an extraordinary emphasis on peripheral processing and global perception, speed in terms of multiple transient events and moving objects and a high degree of perceptual and motor load^30–33,36^. Our findings demonstrate that only the AVG training was able to increase reading speed in children with dyslexia^8,13,33,36^ and only AVG training increased the global before local perception in the computerized Navon task (Fig. 2d,e). In particular, children with dyslexia treated with 12 hours of AVG showed a significant decrease of local interference in the global task and a significant increase of global interference in the local task. A causal connection between local before global perception and reading disabilities is demonstrated, excluding any possible influence of visual-to-phonological access^14^ and indirect phonological^3,5^ or orthographic^28^ stimulation.

Nevertheless, the origin of this lack of a global perception bias observed in unselected and independent samples of children with dyslexia and its remediation by using both reading (RAP)^29^ and non reading (AVG)^8,13,33^ trainings, could be still considered as generated by developmental brain network plasticity after years of deprived reading experience. In particular, a typical reading experience could actively influence visual processing^14^ in developing the common global before local perception found in TR.

In Experiment 5, we longitudinally investigated this question testing a large cohort of pre-literate children and observing their reading development during the next year of the primary school. Future PRs were characterized by a local before global perception at pre-reading stage (Fig. 2f,g). Moreover, independently of our a priori group classification of reading disorder, pre-reading global before local perception was able to predict future efficient reading skills in grade 1 even when chronological age, IQ, visual-to-phonological mapping and pure phonological skills were controlled for. In sum, the global visual perception of preliterate children was linked to reading abilities one year after the first evaluation, demonstrating a causal connection between global before local perception and reading emergency and development.

Similarly to the three independent and unselected groups of children with dyslexia, the future PR children showed visual perceptual skills that seem to proceed without an initial automatic and unskippable global processing^10,22,23,27,28^. This lack of a global perception bias leads to a facilitation in local features extraction^22,23^. Independently from figure sizes, in children with dyslexia and future PRs the local relevant incongruent information appears perceived simultaneously or before the salient global configuration of the visual scene^27^. This peculiar perception leads to a “where is the perceived what” mistake.

The “where” system or magnocellular-dorsal stream - specialized for low spatial frequency processing - could be the underlying neural mechanism of global perception bias. The magnocellular-dorsal stream is specifically involved in the spatial selection and it is impaired in individuals with dyslexia (see^37–39^; see^11^ for review). Magnocellular-dorsal stream functioning as well as reading speed can be efficiently improved in children with dyslexia also using AVG (8, 13, see 36 for review). Recent studies have shown that perceptual learning and transcranial electrical stimulation of magnocellular-dorsal stream are able to improve reading abilities in adults with dyslexia^8,40,41^.

The findings of our 5 experiments are compatible with behavioral, psychophysical, and neuroimaging studies demonstrating the possible role of a right dysfunction of the fronto-parietal network in children and adults with dyslexia (e.g.,^42–45^). Facoetti *et al*.^42^ showed an asymmetric distribution of visual spatial attention in children with dyslexia. In the same vein, Hari *et al*.^43^, in a psychophysical study, showed that adults with dyslexia processed stimuli in the left visual hemifield significantly more slowly than TR, indicating a left-sided mini-neglect, possibly related with a deficit in the right fronto-parietal network. In addition, greater right prefrontal activation during a reading task and greater right superior longitudinal fasciculus white-matter organization significantly predicted future reading outcomes in a longitudinal study on children with dyslexia^44^. The greater functional activation and structural organization of the right fronto-parietal network could indicate a faster global perception of the stimuli^19^ and consequently a greater improvement of reading skills in children with dyslexia^44^. Accordingly, a direct high frequency repetitive transcranial magnetic stimulation over the right parietal cortices improved phonological decoding in adults with dyslexia^46^. A dysfunction in the right parietal network could impair the simultaneous processing that is responsible for poor reading outcomes^10^. TR activate parietal areas more strongly for multiple than single element processing. In contrast, the stronger right parietal areas activation for multiple elements processing was absent in participants with dyslexia (see ref.^47^).

A specific prediction of our findings suggests that a direct cognitive stimulation of the right global perception network by using a remediation Navon program could improve attentional and reading skills. Interestingly, a visual multiple elements training - stimulating the global perception onto whole unit letter string - not only increased bilaterally the activation of the parietal cortices, but also enhanced the fast global reading procedure^10,48^.

Restoring the typical global before local perception through specific trainings of the right fronto-parietal network^27,49^ could improve reading skills in children with reading difficulties, as observed in Experiments 2 and 4. An alerting cue is able to: (i) nullify left-sided neglect in patients with a right fronto-parietal damage^50^; (ii) restore the visual search deficit in children with dyslexia^51^; and (iii) increase global processing bias in adults with ADHD^49^. Several neurodevelopmental disorders show the presence of an interference effect of local-level information on global processing, suggesting that the default processing of people with ADHD and autism spectrum disorder is more local^17,19–21^. Thus, global before local perception trainings could have beneficial effects not only on reading skills, but also on different neurocognitive dysfunctions present in other neurodevelopmental disorders that show this atypical perception of the world.

## Methods

For all the reported experiments, informed written consent was obtained for each child from their parents. The ethic committee of the University of Padua approved the research protocols (n.1451; 1452; 1849). The entire investigation process was conducted according to the principles expressed in the Declaration of Helsinki.

### Experiment 1: Global visual perception in children with dyslexia by using a paper and pencil Navon task

#### Participants

Participants were 180 children from the 2^nd^ to the 5^th^ year of primary school (range 7.2–12 years old; 81 males and 99 female). The sample was collected from 4 Italian schools. All the children were native Italian speakers without any documented history of brain damage, hearing or visual (not corrected) deficits, or ADHD diagnosis^1^.

A series of reading tasks (word and pseudoword lists; see SI) and a Navon stimuli task (Fig. 1a, and SI) were administered in counterbalanced order.

The reading skills of children from the different grades were standardized^52^. Children were divided into two groups: a child was assigned to the group of children with dyslexia if her/his Z score in the mean of speed and accuracy in words and/or pseudowords reading was below −1.5 SDs (n = 17), all the other children were assigned to the TR group (n = 162). The two groups did not differ in chronological age: t_(177)_ = 0.683, p > 0.496 (mean children with dyslexia = 9.17, SD = 1.2 and mean TR = 9.37, SD = 1.14).

### Experiment 2: Global visual perception in children with dyslexia after a visual treatment

#### Participants

Thirteen children (7 female and 6 male) with dyslexia, from the 3^rd^ to 8^th^ school grade (mean age = 10.17 years, SD = 1.87), took part to the visual training based on the reading acceleration program^29^. Children received the diagnosis of dyslexia by the Italian National Health Service, based on standard exclusion and inclusion criteria^1^. The reading performance (errors and/or speed) of each individual was at least −1.5 SDs below the age-standardized norm in at least one of the 4 clinical measures^53^. Other inclusion criteria for this study were normal IQ (≥85), normal or corrected-to-normal vision, absence of neurological deficit and ADHD diagnosis^1^.

A series of reading tasks (word and pseudoword lists), pseudoword repetition, and a Navon stimuli task (Fig. 1a; see Experiment 1) were administered in counterbalanced order (see SI).

### Experiment 3: Global visual perception in children with dyslexia by using a computerized Navon task

#### Participants

Participants were thirty-two children (14 female and 18 male) with dyslexia, from the 3^rd^ to 8^th^ school grade, and fifteen children (11 female and 4 male) TR, from the 1^st^ to 8^th^ school grade. Children with dyslexia were diagnosed by the Italian National Health Service, based on standard exclusion and inclusion criteria^1^. The reading performance (errors and/or speed) of each individual was at least −1.5 SDs below the age-standardized norm in at least one of the 4 clinical measures^53^. Other inclusion criteria for this study were normal IQ (≥85), normal or corrected-to-normal vision, absence of neurological deficit and ADHD diagnosis^1^.

A series of reading tasks (word and pseudoword lists; see SI), and a computerized Navon task (Fig. 2a and SI) were administered in counterbalanced order.

### Experiment 4: Global visual perception in children with dyslexia after an action video game training

#### Participants

Participants were fourteen children (6 female and 8 male; mean age = 10.41 years, SD = 1.71) with dyslexia of Experiment 3 that agreed to be involved to a video game training. A commercial Wii™ video game from Ubisoft™ (deemed suitable for children age 7 and older by the Pan European Game Information) called Rayman Raving Rabbids was used. Single mini-games were selected from the overall game and categorized as AVG or NAVG^13^ (see SI for details). Seven children with dyslexia were assigned to AVG and seven to NAVG training. Reading and phonological skills were similar in the two groups (all ps > 0.392). The two groups did not differ at T1 in both reading (speed and accuracy) and global and local visual perception measurements (all ps > 0.06). Each child was individually treated by playing the commercial Wii™ video game for a total of 12 hours. The single minigames were selected to create the AVG and NAVG trainings^8,13,33^.

Participants were individually tested and trained (see SI) in a dimly lit and quiet room.

A series of reading tasks (word and pseudoword lists; see SI), and a Navon stimuli task (Fig. 2a; see Experiment 3 and SI) were administered in counterbalanced order.

### Experiment 5: Pre-reading global visual perception in future children with reading disorders

#### Participants

Ninety-six (44 female and 52 male) 5-year-old children attending the last year of 4 kindergartens in Northern Italy, took part in the present prospective-longitudinal study. In the Italian school system, formal reading instruction starts in grade 1. Consequently, Italian preschoolers are also pre-readers. We excluded the few children that were able to read at the kindergarten stage and the children with ADHD diagnosis. All children were native Italian speakers without any documented history of brain damage, hearing or visual deficits. The performance IQ level was estimated through the standard scores in block design subtest of the WPPSI scale^34^. The T2 sample was composed by 82 (34 female and 48 male, 14 children moved to other school and become unavailable for testing) children (mean age = 68.5 months, SD = 5.1 and mean Performance IQ = 10.1, SD = 3.5).

#### Computerized Navon task (T1)

The global and local perceptual abilities were measured with the same task used in Experiment 3 and 4, but for each condition 14 trials were presented, for a total amount of 56 trials. Moreover, also the size of stimuli was changed. Geometric figures were shown on a computer screen: A square or a triangle (11.5 × 11.5°) at a global level, which could be formed by small squares or triangles (1.4 × 1.4°) at local level. A small cross (0.2° and 0.6 cd/m^2^) in the centre of the screen served as fixation point.

#### Words text reading task (T2)

Reading fluency and accuracy of a standardized prose passage (140 syllables) was employed to measure ecological-context reading. The speed was measured by total time (in sec) spent on a specific text. Speed and accuracy z-scores were mediated to control reading speed-accuracy trade-off effect.

## Electronic supplementary material

Supplementary information
