# Supplementary material for: A different vision of dyslexia: Local precedence on global perception
Source: Sci Rep. 2017 Dec 12;7:17462. doi: 10.1038/s41598-017-17626-1 (PMC5727118; doi:10.1038/s41598-017-17626-1)
Supplement: Supplementary file 1 — Supplementary information [file 41598_2017_17626_MOESM1_ESM.doc]

**A different vision of dyslexia: Local precedence on global perception**

Sandro Franceschini, Sara Bertoni, Tiziana Gianesini

Simone Gori, and Andrea Facoetti

***Supporting Information (SI)***

**Methods**

In all the experiment, participants were individually tested  (and in Exp. 2 and Exp. 4 also trained) in a dimly lit and quiet room.

Scaled JZS Bayes factors were calculated with the web-based program in http://pcl.missouri.edu.

**Experiment 1: Global visual perception in children with dyslexia by using a paper and pencil Navon task**

The four different conditions composing the Navon stimuli [22] RAN [1] (global vs. local task and congruent vs. incongruent condition) were administered using four paper sheets (Fig. 1a). On each sheet were represented three lines with geometric figures: a triangle, a circle or a square with features that never varied (for example the triangle has always the same characteristics), for a total amount of 7 targets per line (mean distance between the large figures was 4mm). Each global figure (mean height 38mm; mean width 38mm) was composed of smaller local figures (mean height 4mm; mean width 4mm) of the same (congruent) or different (incongruent) stimuli.

In the congruent and incongruent global tasks, children were invited to name aloud the larger figure, independently from the local figures. In contrast, in the congruent and incongruent local tasks, children were invited to name the smaller figure aloud, independently from the global figure. All the children started the tasks from one of the congruent condition (global/local or local/global), and continued with respective incongruent condition (global/local or local/global). The sheets for global and local tasks contained the same figures. Time and errors were measured.

*Reading tasks*

Words reading: the ability to read aloud was measured using words lists composed by 51 words (separated into 3 lists). Words were composed by 2-5 syllables, for a total amount of 149 syllables [52].

Pseudowords reading: phonological decoding ability was measured using two texts, each of 46 pseudowords composed of 1-3 syllables (same syllables in different order for both texts) for a total amount of 100 syllables for each text [52]. Texts order administration was counterbalanced between children. All children were invited to read aloud each text as fast and accurate as possible. Words or pseudowords that were wrongly read, were counted as one error independently from the quantity of wrong letters or syllables pronounced. Self corrections were not classified as errors.

**Results**

We excluded from the analysis children performance more than three boxplot lengths from either end of the box (n=1/180).

Similarly to the original version of RAN tasks [1], mean accuracy was at ceiling (rate=.99), consequently it was not further analyzed.
Main effect of condition and task were significant (F_(1,177)_=85.96, p=.001, η^2^=.327 and F_(1,177)_=17.354, p=.001, η^2^=.089, respectively). The condition x task interaction was significant (F_(1,177)_=6.513, p=.012, η^2^=.035). Group main effect was also significant (F_(1,177)_=7.1, p=.008, η^2^=.039): children with dyslexia group was slower (mean response time=30.42 sec SD=9.28) than TR group (mean response time=25.15 sec SD=7.6).

In the congruent condition, the group with dyslexia was slower than TR group both in global (t_(177)_=-1.755, p=.04 one tile, Cohen’s d=.39, B_01_=1.1) and local (t_(177)_=-3.052, p=.003, Cohen’s d=.69, B_01_=12.99) tasks, whereas in the incongruent condition, the group with dyslexia was slower in comparison to TR group in the global task (t_(177)_=-3.527, p=.001, Cohen’s d=.74, B_01_>30), but not in the local task (t_(177)_=-1.373, p=.172, Cohen’s d=.36, B_01_ in favor of the null= 1.44).

**Experiment 2: Global visual perception in children with dyslexia after a visual treatment**

**Methods**

*Training procedure*

In collaboration with a group of computer scientists and web designers, we created an open access program for pc and tablet that we called “The Library Tower”. Training was structured like a serious game, in which the children have to help an Egyptian magician to save his castle and his books. The application was structured on the basis of a reading acceleration program [29]. The training lasted 10 days distributed across two or three weeks, in daily sessions of about 40 minutes. In each daily session were presented 90 sentences with several lengths (from 18 to 70 characters per sentence), written in white on a black background. Each sentence was presented on a single line. Each sentence was presented only once throughout training and was read silently. A multiple-choice (4 options; chance level=.25) question followed each sentence, asked to recognize the subject or action described in the sentence. Letter by letter erasure time rate (always in left to right reading direction) was determined individually by the mean time necessary to read ten sentences in self-paced silent reading. Erasure time rate was decreased by 3 msec or increased by 2 msec per-letter according to a staircase procedure, based on correct/wrong answer sequences.

*Navon multiple stimuli naming task*

The global and local perception were measured with the same task used in Experiment 1.

*Auditory-phonological task*

Pseudowords repetition task: forty pseudowords (2-5 syllables) were presented to children via headphones [2]. Children had to repeat the single pseudoword acoustically presented. Pseudowords repetition accuracy was measured.

*Words text reading*

Reading speed and errors in age-standardized prose passages from Italian clinical tests [53] were used to measure ecological-context reading.

*Pseudowords reading tasks*

Phonological decoding abilities were measured using a standardized list of 48 Italian pseudowords, for a total amount of 127 syllables [53] and two pseudowords texts [52] (see Experiment 1).

**Results**

*Navon multiple stimuli naming task*

Similarly to the original version of RAN tasks [1] and Experiment 1, mean accuracy in the Navon multiple stimuli naming task, reached ceiling effect (rate=.98) in T1 and consequently was not further analyzed.

The main effects of time (F_(2,24)_=13.318, p=.0001 η^2^=.526), condition (F_(1,12)_=15.007, p=.002 η^2^=.556) and task (F_(1,12)_=6.825, p=.023 η^2^=.363) were significant. Condition x task interaction was also significant (F_(1,12)_=14.392, p=.003 η^2^=.545).

*Training effect on reading skills*

In order to evaluate the effects of RAP training [29] on reading skills, we conducted four different ANOVAs on response time (in sec) and errors (number) as dependent variables, and with time (T1, T2 and T3) as within-subject factor.

Words text reading: the ANOVA on the speed of words text reading revealed a significant effect of time (F_(1.138,13.658)_=4.545, p=.048 η^2^=.275). Paired sample t-test showed that differences were significant only between T2-T3 (see main text) and T1-T3 (T1 mean=343 sec, SD=270; t_(12)_=2.187, p=.049, Cohen’s d=.61, B_01_=1.77; T1-T2 t_(12)_=1.643, p=.126, Cohen’s d=.17, B_01_in favor of the null=1.06). A second ANOVA on errors, showed no significant effects (F_(2,24)_=.926, p=.41, η^2^=.072). Words text reading errors were not influenced by the treatment (T1 mean=12, SD=12; T2 mean=11, SD=11; T3 mean=13, SD=16). The improvement in text reading speed was not explained by a speed/accuracy trade-off effect.

Two months after the end of RAP training (T4), the same group of children with dyslexia was again evaluated in their words text reading abilities: no significant difference between T4 (mean=252 sec, SD=177) and T3 was found in reading speed (t_(12)_=-.799, p=.44, Cohen’s d=.22, B_01_in favor of the null=2.17) or accuracy (t_(12)_=1.484, p=.164 , Cohen’s d=.41, B_01_in favor of the null=1.25; T4 mean=8.68, SD=10.42). These findings showed that the reading improvement was still maintained, demonstrating a long lasting effect of the brief and intensive RAP training.

To confirm the results about reading speed improvement, in T2 and T3, we administered two standardized reading texts. T-test comparisons on reading speed revealed an improvement between T2 (z score mean=-2.12, SD=1.11) and T3 (z score mean=-1.84, SD=.92; t_(12)_=-2.104, p=.03 , Cohen’s d=.28, B_01_=1.59), without changes in reading accuracy from T2 (z score mean=-1.64, SD=1.98) to T3 (z score mean=-1.25, SD=2.74; t_(12)_-.57, p=.579 , Cohen’s d=.17, B_01_in favor of the null=2.42).

Phonological decoding: we found a significant effect of time also for pseudowords reading speed (F_(1.138,16.613)_=6.479, p=.014 η^2^=.351). Paired sample t-test revealed that only T2-T3 (see main text) and T1-T3 (T1 mean=234 sec, SD=87; t_(12)_=2.898, p=0.013, Cohen’s d=.69, B_01_=4.54) were significantly different. Treatment with time constraint significantly improved pseudowords reading speed. Considering errors number as dependent variable, ANOVA results showed no significant changes (F_(2,24)_=.433, p=.653 η^2^=.035) in number of errors across the three evaluations, excluding an effect on accuracy. Neither words text reading nor pseudowords reading accuracy were influenced by the treatment. The reading improvements after the visual training were characterized by the increased reading speed without any cost in accuracy.

**Experiment 3: Global visual perception in children with dyslexia by using a computerized Navon task**

**Methods**

*Participants*

The two groups did not differ for chronological age: t_(45)_=.648, p>.521, Cohen’s d=.19 (mean children with dyslexia= 10.09, SD=1.49 and mean TR=9.73, SD=2.25). The two groups differed both in words reading time (t_(45)_=3.087, p=.003, Cohen’s d=1.1; TR: mean=137.84, SD=98.09; children with dyslexia: mean=291.08, SD=179.42) and errors (t_(45)_=5.131, p=.001, Cohen’s d=1.94; TR: mean=2.27, SD=3.06; children with dyslexia: mean=12.16, SD=7.13), and pseudowords  reading time (t_(45)_=3.004, p=.004, Cohen’s d=1.09; TR: mean=88.32, SD=48.46; children with dyslexia: mean=165.39, SD=93.26) and errors (t_(45)_=4.705, p=.001, Cohen’s d=1.74; TR: mean=3.6, SD=3.91; children with dyslexia: mean=14.09, SD=8.18).

To balance the dimension of the two groups, we added 17 children (TR n=32 , children with dyslexia n=32). The two groups did not differ for chronological age: t_(62)_=.081, p>.936, Cohen’s d=.02 (mean TR=10.06, SD=1.74). The two groups differed both in words reading time (t_(62)_=5.308, p=.0001, Cohen’s d=1.44.; TR: mean=109.06, SD=73.78) and errors (t_(62)_=6.782, p=.0001, Cohen’s d=1.86; TR: mean=3, SD=2.74), and pseudowords  reading time (t_(62)_=5.041, p=.0001, Cohen’s d=1.37; TR: mean=75.81, SD=37.52) and errors (t_(62)_=6.522, p=.0001, Cohen’s d=1.79; TR: mean=4.03, SD=3.05).

*Computerized Navon task*

Participants sat 42 cm away from the pc screen. Geometric figures were shown on a computer screen: a square or a triangle (7.8 x 7.8°) at a global level, which could be formed by small squares or triangles (0.8 x 0.8°) at local level. The experiment included two different tasks, administered in counterbalanced order. Children had to indicate the global or the local figure. Stimuli features were both congruent or incongruent: i) in the congruent condition the global figure had the same shape of local figures (a big triangle composed by little triangles 20 cd/m^2^) and ii) in the incongruent condition, instead, the global figure had a different shape from local figures (a big triangle composed by little squares). A small cross (0.1° and .6 cd/m^2^) in the centre of the screen served as fixation point. Each trial started with a white screen (119 cd/m^2^), after 1500 msec the fixation point appeared for 350 msec, then one of the four possible figures appeared (a square or triangle, made of congruent or incongruent figures) and did not disappear until the children pressed the button (C or M on a keyboard) or max 5 seconds, to indicate the triangle(s) or the square(s), respectively. Each condition consisted of 20 trials, for a total amount of 80 trials.

*Reading tasks*

Words and pseudowords reading tasks: phonological decoding abilities were measured using a standardized list of pseudowords [53]. Reading abilities were measured using a standardized list of 112 Italian words, for a total amount of 218 syllables [53].

**Results**

*Computerized Navon Task*

Mean accuracy (rate=.95) was at ceiling, consequently it was not further analyzed.

Main effect of condition and task were significant (F_(1,45)_=43.48, p=.001, η^2^=.491 and F_(1,45)_=6.113, p=.017, η^2^=.120, respectively).

We added 17 children without any reading difficulties and we re-ran an ANOVA on the Navon global task with a 2 x 2 design. Again, mean accuracy was at ceiling (.97). The within-subject factors was condition (congruent and incongruent), while the between-subject factor was group (children with dyslexia and TR). Main effect of condition (F_(1,62)_=27.994, p=.0001, η^2^=.311) and condition x group interaction were significant (F_(1,62)_=19.214, p=.0001, η^2^=.237). (Fig. 2b)

**Experiment 4: Global visual perception in children with dyslexia after an action video game training**

**Methods**

*Participants*

Information about video game experience were collected during interviews with parents during pre-informative briefing about the experimental training. Children with dyslexia did not know the aim of the training and in the previous six months did not play action any video game (AVG) for more than 1 hour per month.

*Training procedure*

Participants were tested before 3 to 5 days the start of treatment and re-tested between one and three days after the end of training. Video games were played standing 200 cm from a 27-in TV screen. In order to classify the mini-games, we followed the checklist developed by Green et al. [3]. The NAVG participants did not see the mini games used by the AVG players and vice versa. We trained children for 9 sessions of 80 minutes per day distributed across a period of two weeks [8, 13, 33].

*Reading tasks*

Words text reading task [53]: see Experiment 2.

Pseudowords reading tasks: phonological decoding abilities were measured using two pseudowords texts[44], and two lists, of 15 pseudowords each [44] composed of 2-4 syllables (the same syllables in different order for both lists). Pseudowords texts and lists order administration were counterbalanced between children in T1 and T2.

***Supplementary Figure 1****.* Non action video game (NAVG) training effects on global and local Navon task in Experiment 4. After the NAVG training children with dyslexia (D) did not show a significant reduction of local interference effect in the global task (a) or a significant increase of global interference effect in the local task (b).

**Experiment 5: Pre-reading global visual perception in future children with reading disorders**

**Methods**

*Auditory-phonological processing*

Phonological skills at the syllabic level were tested by using one task included in the Italian “Phonological Awareness Battery” [35], that is the “Syllabic blending”, measuring the ability to blend segmented syllables into a word (15 words; e.g., “fi”, “o” and “re”=“fiore” (flower in Italian)). The number of errors was recorded.

*Visual-to-phonological mapping task*

Cross-modal mapping from visual stimuli to the correspondent spoken words (the access to phonological lexicon from the visual input) was measured by using a non-alphabetic RAN task, in which the visual items were 16 filled coloured circles. The task was to name the familiar colours as fast as possible by filling the circles [12]. The dependent variable was the total time (in sec) for naming all the visual items.

**Results**

**Reading and Phonological skills**

*Group analysis*

Children at the end of grade 1 (T2) were divided into PR and TR groups. The two groups were significantly different in text reading skills (PR mean=-2.92, SD=1.1; TR mean=-0.14, SD=.71 t_(80)_=11.89, p=.001, Cohen’s d=3.07; see Franceschini et al. [12] for details). These two groups did not differ for chronological age (PR mean=5.65 years, SD=.53; TR mean=5.73 years, SD=.41 t_(80)_=.624, p=.535, Cohen’s d=.17), performance IQ (PR mean=10.07, SD=2.87; TR mean=10.09, SD=3.69; t_(80)_=.016, p=.987, Cohen’s d=.01), auditory-phonological skills (syllabic blending: PR mean=2.07 errors, SD=1.39; TR mean=1.97 errors, SD=1.63; t_(80)_=-.207, p=.837, Cohen’s d=.07) and visual-to-phonological mapping (RAN of colors: PR mean=19.04 sec, SD=6.4; TR mean=18.67 sec, SD=7.57; t_(80)_=-.168, p=.867, Cohen’s d=.05).

**Computerized Navon task**

Accuracy: only a main effect of condition was significant: F_(1,80)_=9.747, p=.003 η^2^=.109 (congruent condition rate=.95, SD=.07 and incongruent condition rate=.90, SD=.12).

Response times: only a main effect of condition was significant: F_(1,80)_=23.749, p=.0001 η^2^=.229 (congruent condition mean=651 msec, SD=330 and incongruent condition, mean=764 msec, SD=453).

**References**

1. Denckla, M. B. & Rudel, R. G. Rapid “automatized” naming (R.A.N): Dyslexia differentiated from other learning disabilities. *Neuropsychologia* **14**(4), 471–479 (1976).

2. Bertelli, B. & Bilancia, G. Vaumelf-batterie per la valutazione dell’attenzione uditiva e della memoria di lavoro fonologica in età evolutiva. *Bollettino Di Psicologia Applicata* **252**, 74 (2007).

3. Green, C. S., Li, R. & Bavelier, D. Perceptual learning during action video game playing. *Top Cogn Sci* **2**(2), 202–216 (2010).
